# Racemization-free synthesis of *N*α-2-thiophenoyl-phenylalanine-2-morpholinoanilide enantiomers and their antimycobacterial activity

**DOI:** 10.1007/s00726-021-03044-1

**Published:** 2021-07-14

**Authors:** Lea Mann, Markus Lang, Philipp Schulze, Jan Henrik Halz, René Csuk, Sophie Hoenke, Rüdiger W. Seidel, Adrian Richter

**Affiliations:** 1 Institut für Pharmazie, Martin-Luther-Universität Halle-Wittenberg, Wolfgang-Langenbeck-Str. 4, 06120 Halle (Saale), Germany; 2 Max-Planck-Institut für Kohlenforschung, Kaiser-Wilhelm-Platz 1, 45470 Mülheim an der Ruhr, Germany; 3 Institut für Chemie, Martin-Luther-Universität Halle-Wittenberg, Kurt-Mothes Str. 2, 06120 Halle (Saale), Germany

**Keywords:** Amino acid, Phenylalanine amides, MMV688845, GSK1055950A, Racemization-free synthesis, Enantioselective activity, Antimycobacterial activity, Crystal structure

## Abstract

*N*α-2-thiophenoyl-d-phenylalanine-2-morpholinoanilide (MMV688845, IUPAC: *N*-(1-((2-morpholinophenyl)amino)-1-oxo-3-phenylpropan-2-yl)thiophene-2-carboxamide) from the Pathogen Box^®^ library (Medicines for Malaria Ventures, MMV) is a promising lead compound for antimycobacterial drug development. Two straightforward synthetic routes to the title compound starting from phenylalanine or its Boc-protected derivative are reported. Employing Boc-phenylalanine as starting material and the T3P^®^ and PyBOP^®^ amide coupling reagents enables racemization-free synthesis, avoiding the need for subsequent separation of the enantiomers. The crystal structure of the racemic counterpart gives insight into the molecular structure and hydrogen bonding interactions in the solid state. The *R*-enantiomer of the title compound (derived from d-phenylalanine) exhibits activity against non-pathogenic and pathogenic mycobacterial strains, whereas the *S*-enantiomer is inactive. Neither of the enantiomers and the racemate of the title compound shows cytotoxicity against various mammalian cells.

**Supplementary Information:**

The online version contains supplementary material available at 10.1007/s00726-021-03044-1.

## Introduction

Mycobacterial infections are a challenge for anti-infective drug therapy due to a high level of intrinsic and acquired resistance of the pathogens, which usually makes long-time treatments with combined antibiotics necessary (Zumla et al. 2014; To et al. 2020). Rifampicin, an inhibitor of the mycobacterial RNA polymerase with bactericidal activity (Grobbelaar et al. 2019), is a cornerstone for treatment of *Mycobacterium tuberculosis* infections. It belongs to the rifamycin group, the only class of antibiotics with sterilizing activity in caseum (necrotic tissue in granulomas) (Prideaux et al. 2015; Ganapathy et al. 2019). Infections with rifampicin-resistant mycobacteria, e.g. multidrug-resistant *M. tuberculosis* (MDR-Tb) (Stoffels et al. 2013; Dartois et al. 2019; Shah et al. 2020) or *Mycobacterium abscessus* (Nessar et al. 2012; Lopeman et al. 2019), are an arising issue for antimycobacterial chemotherapy. *M. abscessus* infections are particularly difficult to treat, because of a high level of intrinsic resistance against many antitubercular drugs (Nessar et al. 2012; Lee et al. 2015; Bryant et al. 2016). In most cases, patients with a suppressed immune status or a structural lung disease, e.g. cystic fibrosis (Park and Olivier 2015), are affected. Non-tubercular mycobacteria not only cause lung tissue infections (Weiss and Glassroth 2012; Wassilew et al. 2016), but also affect the skin, soft tissue and the central nervous system (Ahmed et al. 2020). Development of new antibiotics is needed to overcome drug resistance and to provide new options for treatment (Wu et al. 2018).

*N*α-2-thiophenoyl-phenylalanine-2-morpholinoanilide (IUPAC: *N*-(1-((2-morpholinophenyl)amino)-1-oxo-3-phenylpropan-2-yl)thiophene-2-carboxamide), the title compound, is a promising lead candidate for anti-mycobacterial drug development (Ballell et al. 2013) with activity against highly drug-resistant non-tubercular mycobacteria, e.g.* M. abscessus* and *Mycobacterium avium* (Ebright et al. 2015; Low et al. 2017; Richter et al. 2018; Jeong et al. 2018). The *R*-enantiomer is contained in the Pathogen Box^®^ library (Medicines for Malaria Venture, MMV, Geneva, Switzerland), as MMV688845. Crystal structures and enzyme inhibition assays with closely related compounds indicate that the activity of *N*α-aroyl-*N*-aryl-phenylalanine amides is caused through inhibition of the RNA polymerase in *M. tuberculosis* (Lin et al. 2017). The clinical importance of RNA polymerase inhibitors mentioned above underlines the suitability of the target for drug development. Since *N*α-aroyl-*N*-aryl-phenylalanine amides interact with a binding site different from that of rifamycins, they have a broader spectrum of activity and there is no cross resistance observed (Lin et al. 2017). MMV688845 was selected for further investigation because it is one of the few RNA polymerase inhibitors that shows significant activity against *M. abscessus*. Thus far, this class of compounds has hardly been studied against non-tuberculous mycobacteria, making it an interesting starting point for further investigation and structural optimisation.

Herein, we report on straightforward synthesis of *N*α-2-thiophenoyl-phenylalanine-2-morpholinoanilide from enantiopure phenylalanine or its Boc-protected derivatives in two steps via two different synthetic routes, one of which leads to racemization, whereas the other preserves a high degree of enantiomeric purity. Both enantiomers of the title compound were synthesized and studied by circular dichroism (CD) spectroscopy and chiral HPLC. The racemic counterpart was structurally characterized by X-ray crystallography. Activities of both enantiomers and the racemate against fast-growing mycobacteria, viz*. M. abscessus* and *Mycobacterium smegmatis*, and their cytotoxicity against mammalian cells were evaluated to gain insight into configurational effects of this compound class.

## Materials and methods

### General

Starting materials were purchased and used as received. Solvents were distilled prior to use and stored over 4 Å molecular sieves. Glassware was oven-dried at 110 °C. Column chromatography was carried out using Merck silica gel 60 (63–200 µm). Flash chromatography was performed on a puriFlash^®^ 430 instrument (Interchim, Montluçon, France). Prepacked columns with silica gel (30 μm) were used. The maximum compound load per column was 5% (m/m) of the silica gel quantity. NMR spectra were recorded on an Agilent Technologies VNMRS 400 MHz spectrometer. Chemical shifts are reported relative to the residual solvent signal (CDCl_3_: *δ*_H_ = 7.26 ppm; *δ*_C_ = 77.36 ppm; CD_3_OD *δ*_H_ = 3.31 ppm; DMSO-*d*6 *δ*_H_ = 2.50 ppm). Abbreviations: *s* singlet, *bs* broad singlet, *d* doublet, *dd* doublet of doublets, *m* multiplet. ESI mass spectra were measured on a Thermo Finnigan LCQ Classic spectrometer and high-resolution mass spectra (HRMS) were recorded on a Thermo Fisher Scientific LTQ Orbitrap XL mass spectrometer. Achiral HPLC analyses were performed using an Agilent 1260 HPLC instrument equipped with UV diode array detection (50 mm Eclipse Plus C18 1.8 µm, 4.6 mm, methanol/water gradient, *v* = 1.0 mL min^−1^, *λ*_used_ = 220 nm), and *ee* determinations were conducted using a Shimadzu Prominence LC-20A HPLC instrument with diode array detection (150 mm Chiralpak IB-N3, 4.6 mm, acetonitrile/water 55:45, *v* = 1.0 mL min^−1^, *λ*_used_ = 220 nm for compound **2**; 150 mm Chiralpak IC-3, 4.6 mm, acetonitrile/water + 0.1% TFA 30:70, *v* = 1.0 mL min^−1^, *λ*_used_ = 220 nm for compound **1a**).

UV spectra were acquired with a Jasco V-770 UV/Vis spectrophotometer, using 10 mm path length cuvettes, and specific rotation with a Jasco P-2000 polarimeter, using 100 mm path length cuvettes. Circular dichroism spectra were measured with a Jasco J-815 spectrometer, using 1 mm path length cuvettes. Differential Scanning Calorimetry (DSC) was carried out on a Mettler Toledo DSC 823e Module instrument using a standard aluminium sample pan.

### Synthesis

#### Method A

##### *N*α-2-thiophenoyl-l-phenylalanine (1a)

2-Thiophenecarboxylic acid (403 mg, 3.15 mmol) and thionyl chloride (1135 µL, 15.6 mmol) were heated under reflux in toluene (16 mL) for 5 h under nitrogen. After cooling to room temperature, the excess of thionyl chloride and toluene was removed under reduced pressure, and the residue was dissolved in acetonitrile. The 2-thiophenecarbonyl chloride so formed was added to a stirred solution of l-phenylalanine (500 mg, 3.0 mmol) and potassium carbonate (1656 mg, 12.0 mmol) in acetonitrile/water (4:1, 10 mL) at 0° C. The mixture was stirred for 4 h at room temperature. Subsequently, the solvents were removed under reduced pressure and hydrochloric acid (36%) was added to the residue, followed by extraction with diethyl ether (3 × 20 mL). The combined organic layers were dried over sodium sulfate and the solvent was removed under reduced pressure. The crude product was purified by column chromatography eluting with ethyl acetate/heptane (1:1) + 1% formic acid. Yield: 594 mg (2.16 mmol, 72%; HPLC purity 94.7%, *t*_R_ = 19.2 min, 99.1% *ee*) colourless, glass-like substance. ^1^H NMR (400 MHz, CD_3_OD): *δ* = 7.66 (dd, Ar-**H**, *J* = 3.7, 1.3 Hz, 1H), 7.63–7.56 (m, Ar-**H**, 1H), 7.29–7.21 (m, Ar-**H**, 4H), 7.21–7.15 (m, Ar-**H**, 1H), 7.11–7.04 (m, Ar-**H**, 1H), 4.86–4.79 (m, C**H**-CH_2_, 1H), 3.33 (dd, CH-C**H**_**2**_, *J* = 13.9, 5.3 Hz, 1H), 3.11 (dd, CH-C**H**_**2**_, *J* = 13.9, 9.2 Hz, 1H) ppm. HRMS (ESI^+^) m/z [M+H]^+^ calcd for C_14_H_14_NO_3_S^+^, 276.0690 found, 276.0690. Specific rotation, $[ \alpha ]_{{\text{D}}}^{25}$ − 48.4 (*c* 0.130, MeOH).

##### *rac-N*α-2-thiophenoyl-phenylalanine-2-morpholinoanilide (*rac*-2)

Compound **1a** (100 mg, 0.55 mmol), PyBOP^®^ (314 mg, 0.61 mmol) and diisopropylethylamine (153 µL, 0.88 mmol) were dissolved in dichloromethane (10 mL). After addition of 2-morpholinoaniline (100 mg, 0.55 mmol), the reaction mixture was stirred overnight. Solvents were then removed under reduced pressure and the crude product was purified by column chromatography with ethyl acetate/heptane (1:1) followed by washing with heptane/chloroform (95:5). Yield: 125 mg (0.29 mmol, 52%; HPLC purity 98.8%, *t*_R_ = 4.3 min), white solid, m.p. 181–182 °C. ^1^H NMR (400 MHz, CDCl_3_): *δ* = 8.87 (s, N**H**, 1H), 8.38 (d, Ar-**H**, *J* = 8.1 Hz, 1H), 7.54 (dd, Ar-**H**, *J* = 3.8, 1.3 Hz, 1H), 7.51 (dd, Ar-**H**, *J* = 5.0, 1.1 Hz, 1H), 7.33–7.26 (m, Ar-**H**, 4H), 7.24–7.05 (m, Ar-**H**, 5H), 6.78 (s, N**H**, 1H), 4.94 (m, C**H**-CH_2_, 1H), 3.63 (bs, C**H**_**2**_-O-C**H**_**2**_, 4H), 3.38 (dd, CH-C**H**_**2**_, *J* = 13.6, 5.4 Hz, 1H), 3.22 (dd, CH-C**H**_**2**_, *J* = 13.6, 8.0 Hz, 1H), 2.64 (m, C**H**_**2**_-N-C**H**_**2**_, 4H) ppm. ^13^C NMR (101 MHz, CDCl_3_): *δ* = 168.9, 161.7, 141.1, 138.1, 136.6, 133.0, 130.9, 129.5, 129.0, 128.8, 128.0, 127.3, 126.1, 124.6, 120.9, 120.0, 67.3, 56.3, 52.7, 38.7, HRMS (ESI^+^) *m*/*z* [M+H]^+^ calcd for C_24_H_26_N_3_O_3_S^+^, 436.1690; found, 436.1689; [M+Na]^+^ calcd for C_24_H_25_N_3_O_3_SNa^+^, 458.1509; found: 458.1508.

#### Method B

##### Boc-l-phenylalanine-2-morpholinoanilide (*S*-1b)

A solution of Boc-l-phenylalanine (372 mg, 1.4 mmol) and 2-morpholinoaniline (231 mg, 1.3 mmol) in ethyl acetate/pyridine (2:1, 10 mL) and cooled to − 20 °C (isopropanol-dry ice bath), and a solution of T3P^®^ in ethyl acetate (50%, 1.67 mL, 2.8 mmol) was added in small portions. The reaction mixture was stirred overnight at 0 °C in an ice bath. Subsequently, ethyl acetate (20 mL) was added and the mixture was washed with 0.5 N hydrochloric acid (1 × 30 mL). The organic layer was dried over sodium sulfate, and the solvent was removed under reduced pressure. The crude product was purified by flash chromatography eluting with ethyl acetate/heptane. Yield: 476 mg (1.12 mmol, 80%; HPLC purity 99.9%, *t*_R_ = 14.2 min), white solid, m.p. 158–159 °C. ^1^H NMR (400 MHz, CDCl_3_): *δ* 8.93 (s, N**H**, 1H), 8.41 (d, Ar-**H**, *J* = 8.1 Hz, 1H), 7.31–7.02 (m, Ar-**H**, 8H), 5.09 (s, N**H**, 1H), 4.58–4.40 (m, C**H**-CH_2_, 1H), 3.76–3.58 (m, C**H**_**2**_-O-C**H**_**2**_, 4H), 3.18 (dd, CH-C**H**_**2**_, *J* = 11.8, 2.5 Hz, 1H), 3.17 (dd, CH-C**H**_**2**_, J = 11.8, 3.1 Hz 1H), 2.72–2.51 (m, C**H**_**2**_-N-C**H**_**2**_, 4H), 1.41 (s, C(C**H**_**3**_)_3_, 9H) ppm. HRMS (ESI^+^): m/z [M+H]^+^ calcd for C_24_H_32_N_3_O_4_^+^, 426.2388; found: 426.2381. Specific rotation, $[ \alpha ]_{{\text{D}}}^{25}$ − 21.1 (*c* 0.107, MeOH).

##### *N*α-2-thiophenoyl-l-phenylalanine-2-morpholinoanilide (*S*-2)

Compound *S*-**1b** (100 mg, 0.24 mmol) was dissolved in dichloromethane/trifluoroacetic acid (1:1) and stirred for 1 h at room temperature. The solvent was removed under reduced pressure, and the crude product was co-evaporated successively thrice with toluene and thrice with chloroform. The residue was dissolved in dichloromethane followed by addition of diisopropylethylamine (68 µL, 0.72 mmol) and 2-thiophenecarboxylic acid (31 mg, 0.24 mmol). A solution of PyBOP^®^ (135 mg, 0.26 mmol) in dichloromethane was added under light protection and argon. After stirring in the dark overnight, the reaction mixture was washed twice with 0.5 N hydrochloric acid. The organic layer was dried over dried over sodium sulfate and the solvent was removed under reduced pressure. The crude product was purified by flash chromatography eluting with ethyl acetate/heptane. Yield: 32 mg (0.07 mmol, 31%; HPLC purity 98.5%; *t*_R_ = 4.3 min, 99.9% *ee*) colourless, glass-like substance. ^1^H NMR (400 MHz, CDCl_3_): *δ* = 8.82 (s, N**H**, 1H), 8.38 (d, Ar-**H**, *J* = 8.1 Hz, 1H), 7.57–7.46 (m, Ar-**H**, 2H), 7.33–7.26 (m, Ar-**H**, 4H), 7.24–7.04 (m, Ar-**H**, 5H), 6.70 (d, N**H**, *J* = 7.7 Hz, 1H), 4.94 (m, C**H**-CH_2_, 1H), 3.61 (bs, C**H**_**2**_-O-C**H**_**2**_, 4H), 3.39 (dd, CH-C**H**_**2**_, *J* = 13.6, 5.4 Hz, 1H), 3.23 (dd, CH-C**H**_**2**_, *J* = 13.6, 8.1 Hz, 1H), 2.63 (m, C**H**_**2**_-N-C**H**_**2**_, 4H), APT ^13^C NMR (101 MHz, CDCl_3_): *δ* = 168.9, 161.7, 141.3, 138.1, 136.6, 133.0, 131.0, 129.5, 129.0, 128.8, 127.9, 127.3, 125.8, 124.5, 120.9, 119.7, 67.3, 56.3, 52.6, 38.6 ppm. HRMS (ESI^+^): *m*/*z* [M+H]^+^ calcd for C_24_H_26_N_3_O_3_S^+^, 436.1695; found: 436.1686; [M+Na]^+^ calcd. for C_24_H_25_N_3_O_3_SNa^+^, 458.1509; found: 458.1507. Specific rotation, $[ \alpha ]_{{\text{D}}}^{25}$ − 51.5 (*c* 0.200, MeOH).

##### Boc-d-phenylalanine-2-morpholinoanilide (*R*-1b)

A solution of Boc-d-phenylalanine (1000 mg, 3.7 mmol) and 2-morpholinoaniline (611 mg, 3.4 mmol) in ethyl acetate/pyridine (2:1, 15 mL) was cooled to − 20 °C in an isopropanol-dry ice bath, and a solution of T3P^®^ in ethyl acetate (50%, 4.05 mL, 6.8 mmol) was added in several portions. The reaction mixture was stirred overnight at 0 °C. Subsequently, ethyl acetate (30 mL) was added, and the mixture was washed with 0.5 N hydrochloric acid (1 × 30 mL). The organic layer was dried over sodium sulfate, and the solvent was removed under reduced pressure. The crude product was purified by column chromatography eluting with ethyl acetate/heptane. Yield: 1398 mg (3.22 mmol, 95%; HPLC purity 99.9%, *t*_R_ = 14.2 min), white solid, m.p. 155–156 °C. ^1^H NMR (400 MHz, DMSO-*d*6): *δ* = 9.38 (s, N**H**, 1H), 8.21 (d, Ar-**H**, *J* = 7.8 Hz, 1H), 7.47 (d, Ar-**H**, *J* = 8.3 Hz, 1H), 7.34–7.16 (m, Ar-**H**, 6H), 7.16–7.04 (m, Ar**H** + N**H**, 2H), 4.42–4.31 (m, C**H**-CH_2_, 1H), 3.88–3.68 (m, C**H**_**2**_-O-C**H**_**2**_, 4H), 3.24 (dd, CH-C**H**_**2**_, *J* = 13.8, 4.5 Hz, 1H), 2.87–2.75 (m, C**H**_**2**_-N-C**H**_**2**_ + CH-C**H**_**2**_, 3H), 2.72–2.66 (m, C**H**_**2**_-N-C**H**_**2**_, 2H), 1.30 (s, C(C**H**_**3**_)_3_, 9H) ppm. HRMS (ESI^+^): m/z [M+H]^+^ calcd for C_24_H_32_N_3_O_4_^+^, 426.2388; found: 426.2381. Specific rotation, $[ \alpha ]_{{\text{D}}}^{25}$ + 21.7 (*c* 0.307, MeOH).

##### *N*α-2-thiophenoyl-d-phenylalanine-2-morpholinoanilide (*R*-2)

Compound *R*-**1b** (250 mg, 0.59 mmol) was dissolved in dichloromethane/trifluoroacetic acid (1:1) and stirred for 1 h at room temperature. The solvent was removed under reduced pressure and the crude product was co-evaporated successively thrice with toluene and thrice with chloroform. The residue was dissolved in dichloromethane followed by the addition of diisopropylethylamine (308 µL, 1.77 mmol) and 2-thiophenecarboxylic acid (75 mg, 0.59 mmol). A solution of PyBOP^®^ (338 mg, 0.65 mmol) was added under light protection and argon. After stirring overnight, the reaction mix was washed twice with 0.5 N hydrochloric acid. The organic layer was dried over sodium sulfate, and the solvent was removed under reduced pressure. The crude product was purified by flash chromatography eluting with ethyl acetate/heptane. Yield: 204 mg (0.47 mmol, 80%; HPLC purity 99.0%, *t*_R_ = 4.3 min, 99.9% *ee*) colourless, glass-like substance. ^1^H NMR (400 MHz, CDCl_3_): *δ* = 8.80 (s, N**H**, 1H), 8.40 (dd, Ar-**H**, *J* = 8.1, 1.5 Hz, 1H), 7.52 (dd, Ar-**H**, *J* = 3.8, 1.1 Hz, 1H), 7.50 (dd, Ar-**H**, *J* = 5.0, 1.1 Hz, 1H), 7.32–7.25 (m, Ar-**H**, 4H), 7.24–7.04 (m, Ar-**H**, 5H), 6.81 (d, N**H**, *J* = 7.7 Hz, 1H), 4.94 (m, C**H**-CH_2_, 1H), 3.68–3.51 (m, C**H**_**2**_-O-C**H**_**2**_, 4H), 3.39 (dd, CH-C**H**_**2**_, *J* = 13.6, 5.4 Hz, 1H), 3.23 (dd, CH-C**H**_**2**_, *J* = 13.6, 8.0 Hz, 1H), 2.70–2.49 (m, C**H**_**2**_-N-C**H**_**2**_, 4H) ppm. APT ^13^C NMR (101 MHz, CDCl_3_): *δ* = 168.8, 161.6, 141.3, 138.1, 136.6, 133.0, 130.9, 129.5, 129.0, 128.8, 128.0, 127.3, 126.0, 124.5, 120.9, 119.6, 67.4, 56.3, 52.6, 38.7 ppm. HRMS (ESI^+^): *m*/*z* [M+H]^+^ calcd for C_24_H_26_N_3_O_3_S^+^, 436.1690; found: 436.1690; [M+Na]^+^ calcd for C_24_H_25_N_3_O_3_SNa^+^, 458.1509; found: 458.1506. Specific rotation, $[ \alpha ]_{{\text{D}}}^{25}$ + 55.2 (*c* 0.240, MeOH).

### X-ray crystallography

A crystal of *rac*-**2** suitable for single-crystal X-ray diffraction was obtained from a solution in methanol/water by slow evaporation of the solvents at ambient conditions. The X-ray intensity data were collected on a Stoe IPDS II diffractometer using graphite-monochromated Mo-K_α_ radiation. The crystal structure was solved with SHELXT (Sheldrick 2015b) and refined with SHELXL-2018/3 (Sheldrick 2015a). Carbon-bound hydrogen atoms were placed in geometrically calculated positions and refined with an appropriate riding model. Hydrogen atoms bonded to nitrogen were located via difference Fourier syntheses and refined freely. Structure pictures were drawn with Diamond (Brandenburg 2018) and Mercury (Macrae et al. 2020). The packing index was calculated with Platon (Spek 2009).

Crystal data for *rac*-**2**: C_24_H_25_N_3_O_3_S, *M*_r_ = 435.53, *T* = 170(2) K, *λ* = 0.71073 Å, monoclinic, space group *P*2_1_/*n*, *a* = 9.6017(4), *b* = 9.8739(3), *c* = 23.2482(12) Å, *β* = 101.851(4)°, *V* = 2157.09(16) Å^3^, *Z* = 4, *ρ*_calc_ = 1.341 mg m^−3^, *μ* = 0.182 mm^−1^, *F*(000) = 920, crystal size = 0.165 × 0.152 × 0.131 mm, *θ* range = 1.79—27.41°, reflections collected/unique = 13,647/4846 (*R*_int_ = 0.0557), 288 parameters, *S* = 1.017, *R*1 [*I* > 2*σ*(*I*)] = 0.0449, *wR*2 = 0.1110, *Δρ*_max_, *Δρ*_min_ = 0.281, −  0.322 eÅ^−^^3^.

### Microbiological assays

MIC determination against *M. smegmatis* mc^2^ 155 pTEC27 and *M. abscessus* ATCC 19977 pTEC27. MICs were determined by the broth microdilution method. 96-well flat bottom tissue culture plates (Sarstedt, 83.3924.500) were used (Richter et al. 2018). In the second well of each row, two times of the desired highest concentration of each compound was added in 7H9 medium supplemented with 10% ADS and 0.05% polysorbate 80. Each compound was diluted twofold in a ten-point serial dilution. The concentration of the starting inoculum was 5 × 10^5^ cells mL^−1^. The starting inoculum was diluted from a pre-culture at the mid-log phase (OD_600_ 0.3 to 0.7) and an OD_600_ of 0.1 was correlated to 1 × 10^8^ CFU mL^−1^. The plates were sealed with parafilm, placed in a container with moist tissue and incubated for four days at 37 °C. Each plate had eight negative controls (1% dimethylsulfoxide) and eight positive controls (100 µM amikacin). After incubation, the plates were monitored by OD measurement at 590 nm (Tecan SpectraFluor). The assay was performed in duplicate and results were validated by RFP measurement. Data analysis: Every assay plate contained eight wells with dimethylsulfoxide (1%) as negative control, which corresponds to 100% bacterial growth and eight wells with amikacin (100 µM) as positive control in which 100% inhibition of bacterial growth was reached. Controls were used to monitor the assay quality through determination of the Z′ score. The Z′ factor was calculated as follows:

$$Z^{\prime} = 1 - \frac{{3\left( {{\text{SD}}_{{{\text{amikacin}}}} + {\text{SD}}_{{{\text{DMSO}}}} } \right)}}{{M_{{{\text{amikacin}}}} - M_{{{\text{DMSO}}}} }}$$

SD is the standard deviation, and *M* is the mean.

The percentage of growth inhibition was calculated by the equation:

$$\% { }\;{\text{Growth }}\;{\text{inhibition}} = - 100 \% \times \frac{{{\text{OD}}_{590} \;({\text{sample}}) - {\text{OD}}_{590} \;({\text{DMSO}})}}{{{\text{OD}}_{590} \;({\text{DMSO}}) - {\text{OD}}_{590} \;({\text{amikacin}})}}$$

### Cytotoxicity assay

Human tumor cell lines A375 (melanoma), HT29 (colon adenocarcinoma), MCF-7 (breast adenocarcinoma), A2780 (ovarian carcinoma) and non-malignant mouse fibroblasts NIH 3T3 were used. The cell lines were obtained from the Department of Oncology, Martin-Luther-Universität Halle-Wittenberg (Halle (Saale), Germany). Cultures were maintained as monolayers in RPMI 1640 medium with l-glutamine (Capricorn Scientific GmbH, Ebsdorfergrund, Germany) supplemented with 10% heat inactivated fetal bovine serum (Sigma-Aldrich Chemie GmbH, Steinheim, Germany) and penicillin/streptomycin (Capricorn Scientific GmbH, Ebsdorfergrund, Germany) at 37 °C in a humidified atmosphere with 5% carbon dioxide.

The cytotoxicity of the compounds was evaluated using the sulforhodamine B (SRB, Kiton-Red S, ABCR) micro-culture colorimetric assay. Cells were seeded into 96-well plates on day 0. After 24 h, the cells were treated with different concentrations of the compounds to be tested. The final concentration of dimethylsulfoxide/dimethylformamide never exceeded 0.5%, which was non-toxic to the cells. After a 72-h treatment, the supernatant medium from the 96-well plates was discarded, the cells were fixed with 10% trichloroacetic acid and allowed to rest at 4 °C. After 24 h fixation, the cells were washed in a strip washer and dyed with SRB solution (100 µL, 0.4%, in 1% acetic acid) for about 20 min. After dying, the plates were washed four times with 1% acetic acid to remove the excess of the dye and allowed to air-dry overnight. TRIS base solution (200 µL, 10 mM) was added to each well and the absorbance was measured at *λ* = 570 nm using a 96-well plate reader (Tecan Spectra, Crailsheim, Germany). The EC_50_ values were averaged from three independent experiments performed each in triplicate calculated from semi-logarithmic dose–response curves applying a non-linear 4P Hills-slope equation (GraphPad Prism5; variables top and bottom were set to 100 and 0, respectively).

## Results and discussion

### Synthesis

**Scheme 1:**
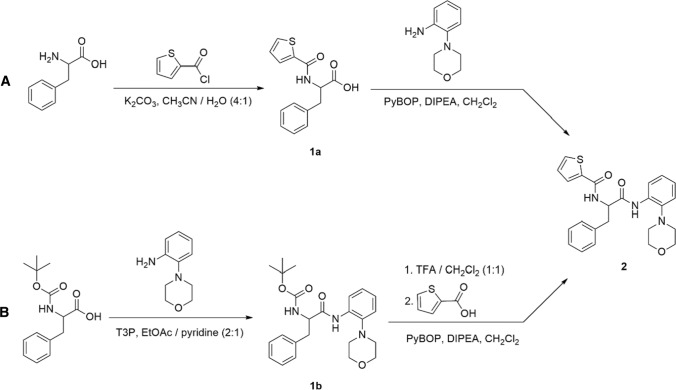
Synthesis of **2**. Method A: *N*-acylation of d- or l-phenylalanine with 2-thiophenecarbonyl chloride followed by amide coupling with 2-morpholinoanilin. Method B: Amide coupling of d- or l-Boc-phenylalanine with 2-morpholinoaniline followed by Boc deprotection and amide coupling with 2-thiophenecarboxylic acid. PyBOP^*®*^ (benzotriazolyloxy-tris[pyrrolidino]-phosphonium hexafluorophosphate), DIPEA (diisopropylethylamine), T3P^*®*^ (propanephosphonic acid anhydride), TFA (trifluoroacetic acid)

**Fig. 1:**
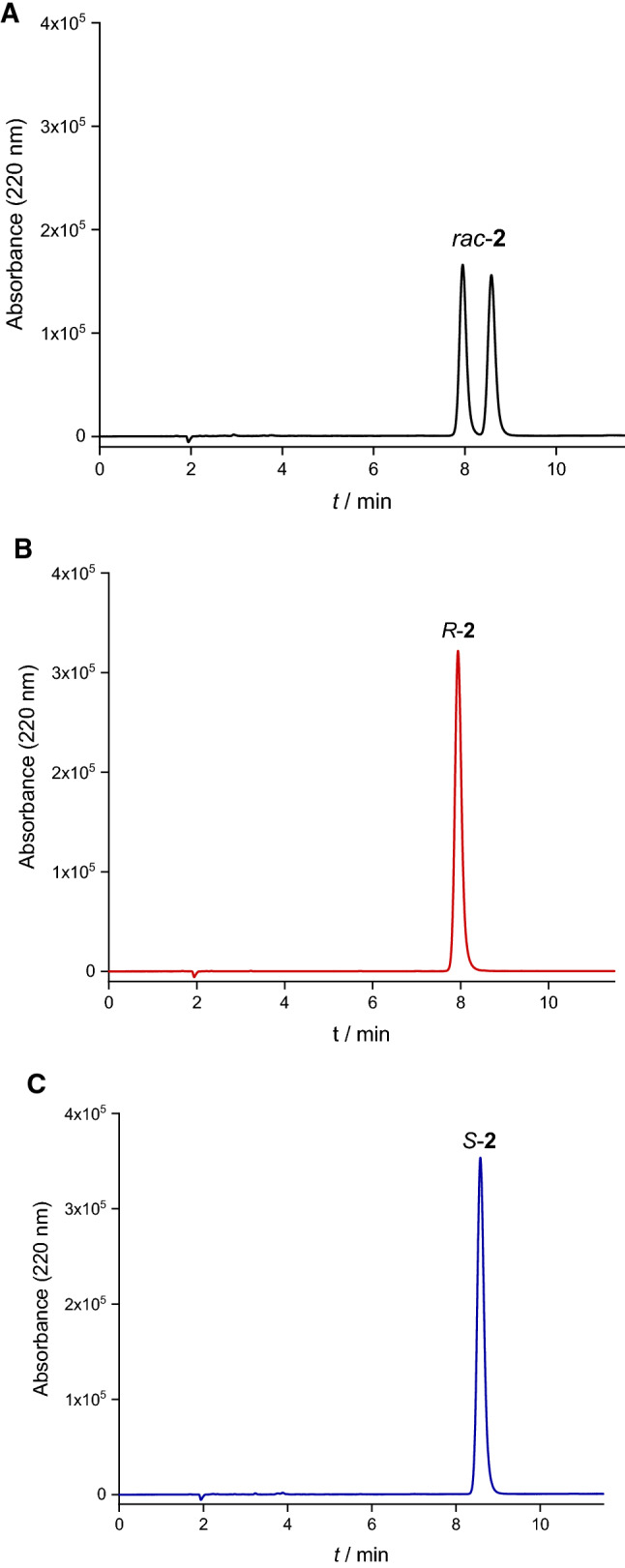
Chiral HPLC chromatograms of *rac*-**2** (**A**), *R*-**2** (**B**) and *S*-**2** (**C**) (Chiralpak IB-N3, acetonitrile/water 55:45)

For the synthesis of the target compound **2**, two different synthetic routes were investigated (Scheme 1). Method A started with the unprotected amino acid l-phenylalanine, which was reacted with 2-thiophenecarbonyl chloride to afford the amide coupling product *N*α-2-thiophenoyl-phenylalanine (**1a**) in accordance with the literature (Draper et al. 2015; Skogh et al. 2017). In the second step, **1a** was coupled with 2-morpholinoaniline using PyBOP^®^ as coupling reagent (Coste et al. 1990) in the presence of diisopropylethylamine (Hünig’s base) to give the targeted **2** in good yield. X-ray crystallography first revealed the formation of a racemic product, which was confirmed for the bulk sample by the absence of specific rotation and chiral HPLC (Fig. 1). The compound also showed no chiroptical signal in the CD spectrum. The racemisation occurs during the conversion of **1a** to *rac*-**2** using PyBOP^®^ (Coste et al. 1990), as the intermediate **1a** still has an *ee* of 99.1% which was detected by chiral HPLC (Figure S10). The presence of a specific rotation signal also indicates that **1a** is not racemic. During the conversion to *rac*-**2**, the carboxy group is activated as a hydroxybenzotriazol ester, which increases the electron withdrawing effect on the chiral centre at the α-carbon atom of the amino acid and accounts for the observed racemisation in the presence of diisopropylethylamine. As expected, applying method A for d-phenylalanine as starting material likewise afforded *rac*-**2**. The crystal structure of *rac*-**2** is described in “Structural description of *rac*-**2**”.

**Fig. 2:**
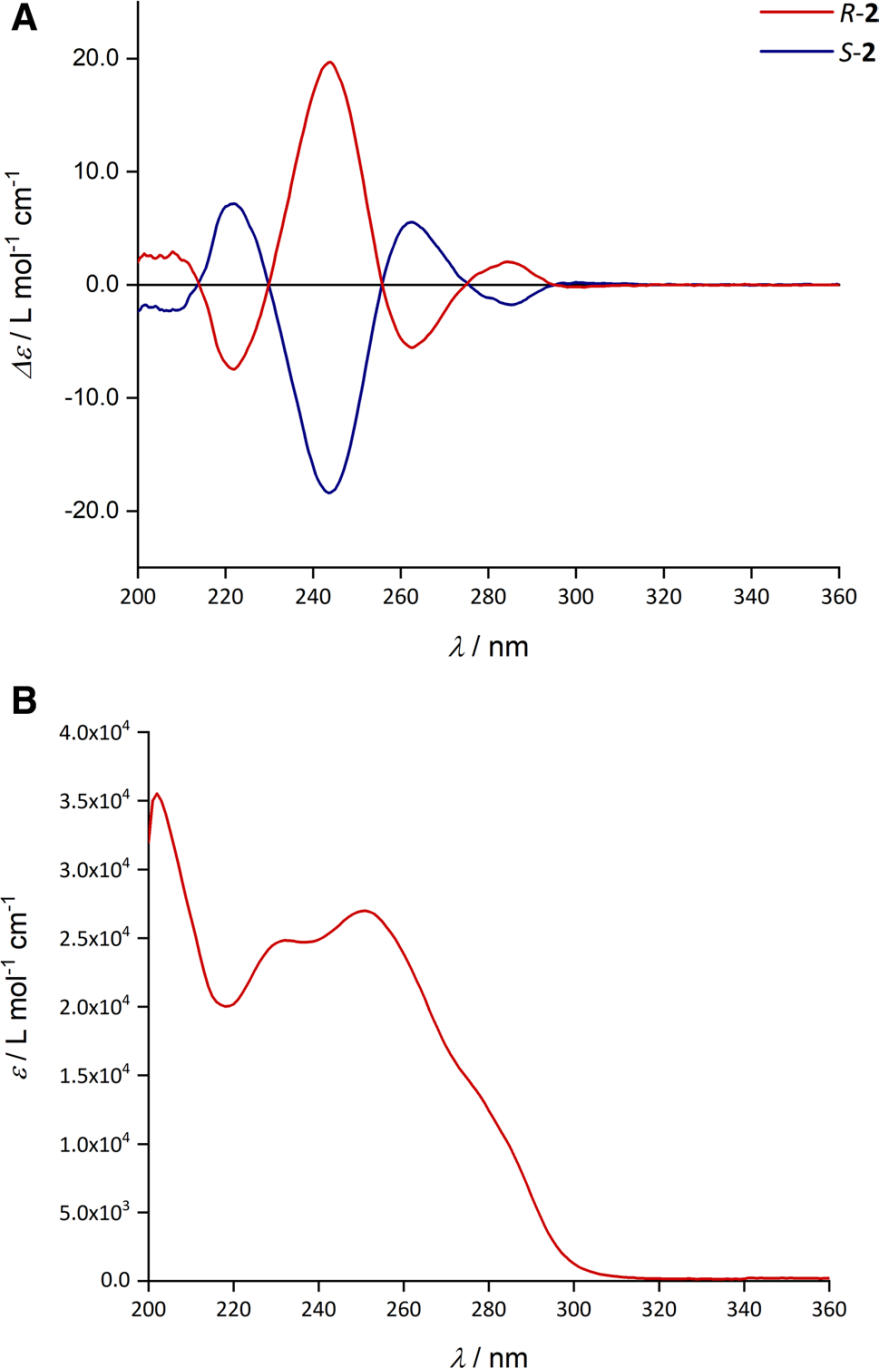
**A** CD spectra of *R*-**2** (*c* = 5.51 × 10^−4^ mol L^−1^) and *S*-**2** (*c* = 4.59 × 10^−4^ mol L^−1^) in methanol. **B** UV spectrum of *R*-**2** in methanol (*c* = 9.18 × 10^−6^ mol L^−1^). UV spectra of *S*-**2** and *rac*-**2** in methanol can be found in the Supplementary Material

In method B, starting with Boc-protected d- or l-phenylalanine, amide bond-formations were achieved with the aid of the coupling reagents T3P^®^ (Wissmann and Kleiner 1980) and PyBOP^®^, which are known from peptide synthesis for a low degree of racemization or epimerization (Dunetz et al. 2011; Pelay-Gimeno et al. 2013; Waghmare et al. 2014). Coupling with 2-morpholinoaniline was carried out using T3P^®^ in ethyl acetate/pyridine (2:1) at 0 °C for conservation of the configuration at the asymmetric α-carbon atom of the amino acid. After isolation of **1b**, the Boc-protecting group was removed with trifluoroacetic acid to obtain d- or l-phenylalanine-2-morpholinoanilide, which was reacted in situ with 2-thiophenecarboxylic acid, using PyBOP^®^ and diisopropylethylamine. Specific rotation measurements and CD spectroscopy proved the formation of optically active products. Both enantiomers, viz*. R*-**2** obtained from d-phenylalanine and *S*-**2** derived from l-phenylalanine, show equal but opposite CD signals in their electronic absorption range of 200–300 nm, as expected (Fig. 2). Chiral HPLC revealed 99.9% *ee* for both *R*- and *S*-**2** (Fig. 1).

Despite intense efforts using different solvent systems for crystallization, single-crystals of *R*-**2** and *S*-**2** suitable for X-ray diffraction could not be obtained. All attempts resulted in the formation of glassy materials. For *R*-**2** isolated from chloroform, the amorphous nature of the solid was confirmed by a DSC scan, which revealed a glass transition at 45–52 °C (see Supplementary Material). Packing difficulties due to an awkward molecular shape can be put forward to explain inability of *R*- and *S*-**2** to form crystalline solids. Clearly, the formation of centrosymmetric hydrogen-bonded dimers, as encountered in the crystal structure of the racemic counterpart (*rac*-**2**, see “Structural description of *rac*-**2**”) is impossible for enantiopure **2**.

### Structural description of *rac*-2

**Fig. 3:**
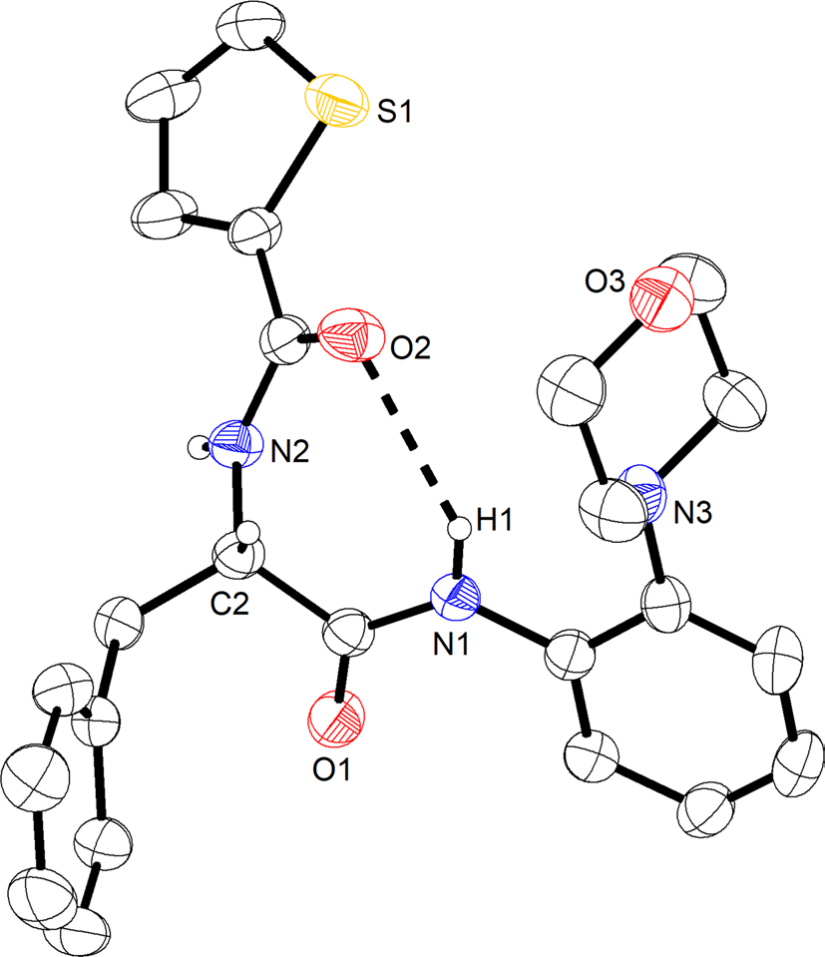
Molecular structure of the *S*-enantiomer in the chosen asymmetric unit of the centrosymmetric crystal structure of *rac*-**2**. Displacement ellipsoids are drawn at the 50% probability level, and hydrogen atoms are represented by small spheres of arbitrary radius. Those attached to carbon are omitted for clarity, except for the asymmetric carbon atom C2. The dashed line represents a hydrogen bond: N1–H1 = 0.88(2), H1⋯O2 = 2.45(2), N1⋯O2 = 3.158(2) Å, N1–H1⋯O2 = 138(2)°

Solvent-free *rac*-**2** was found to crystallize in the centrosymmetric monoclinic space group *P*2_1_/*n* with four molecules in the unit cell. Figure 3 shows the molecular structure of the *S*-enantiomer (corresponding to the l form) in the chosen asymmetric unit of *rac*-**2**. As mentioned above, the molecule exhibits a somewhat awkward irregular shape. Both amide groups adopt a *Z* conformation. The twist angle *τ* is 173.7° for the 2-morpholinoanilide group and − 170.3° for the *N*α-2-thiophenoyl amide group, indicating some out-of-plane deformation of the amide linkages (Winkler and Dunitz 1971; Yamada 2000). The pyramidality at the amide N atoms *χ*_N_ (Yamada 2000), according to Winkler and Dunitz (1971), is small at − 1.9° and − 2.5° for N1 and N2, respectively, and *χ*_C_ is close to zero in both amide groups. N1 of the 2-morpholinoanilide group forms an intramolecular hydrogen bond to O2 of the thiophenoyl group with a S(7) motif (Bernstein et al. 1995). The mean plane calculated through the morpholine saturated six-membered ring is inclined to the mean plane of the aniline aromatic ring by ca. 72°. The morpholine ring adopts a low-energy chair conformation, and the coordination at the morpholine nitrogen atom N3 is markedly pyramidal. Structurally characterized examples of 2-morpholinoanilides are surprisingly rare. A search of the Cambridge Structural Database [CSD; version 5.41 with August 2020 updates (Groom et al. 2016)] revealed only two structures, viz*.* the 2-morpholinoanilides of picolinic acid (CSD refcode: HOBQEW) and pyrimidine-4-carboxylic acid (HOBQAS) (Li et al. 2014), in which the 2-morpholinoanilide moiety shows a similar structure as in *rac*-**2**.

**Fig. 4:**
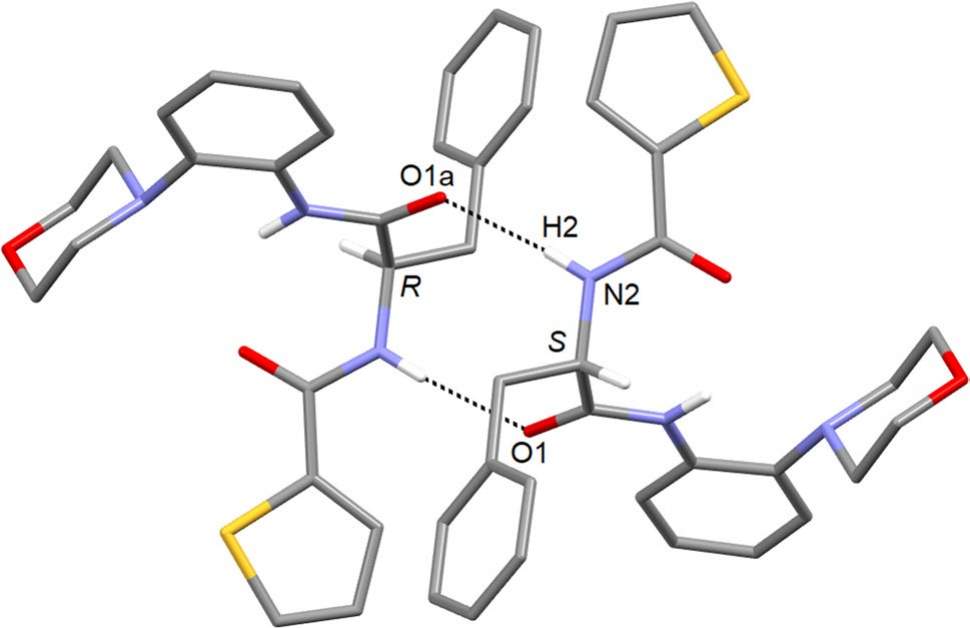
Centrosymmetric dimer in the crystal structure of *rac*-**2** with a ${\text{R}}_{2}^{2} (10)$ hydrogen bond motif. Carbon-bound hydrogen atoms are omitted for clarity, except for the asymmetric carbon atom. The dashed line represents hydrogen bonds: N–H1 = 0.82(2), H1⋯O2 = 2.11(3), N1⋯O2 = 2.925(2) Å, N1–H1⋯O2 = 174(2)°. Symmetry code: (a) − *x* + 1, − *y* + 1, − *z* + 1

In the crystal, the molecules form a centrosymmetric dimer about a crystallographic center of symmetry with a ${\text{R}}_{2}^{2} (10)$ hydrogen bond motif, involving N2 as hydrogen bond donor and O2 as acceptor (Fig. 4). The hydrogen bonding parameters for *rac*-**2** are within expected ranges (Thakuria et al. 2017), and the observed hydrogen bonding scheme is in line with Etter’s third rule for hydrogen bonding, which states that the best hydrogen bond donors and acceptors remaining after intramolecular hydrogen bond formation form intermolecular hydrogen bonds to one another (Etter 1990). The calculated packing index of 69.1% is typical of molecular crystals (Kitajgorodskij 1973).

### Biological evaluation of *rac*-2, *R*-2 and *S*-2

Anti-mycobacterial activities of racemic and enantiopure **2** were determined against two species of fast-growing mycobacteria, viz*. M. abscessus* ATCC 19977 and *M. smegmatis* mc2 155, in a microplate dilution assay (Richter et al. 2018). The results are shown in Figure S9 in the Supplementary Material. Against *M. abscessus*, a pathogenic and rifampicin-resistant bacterium, *R*-**2** possesses an MIC_90_ of 6.25 µM, which is consistent with the literature (Low et al. 2017; Richter et al. 2018). In contrast, *S*-**2** does not show activity against these two mycobacterial species, and *rac*-**2** causes growth inhibition up to a concentration of 25 µM. Against *M. smegmatis*, which is generally considered non-pathogenic (Joseph Antony Sundarsingh et al. 2020), lower MICs than those against *M. abscessus* were observed for *R*-**2** and *rac*-**2** (Table 1). Likewise, *S*-**2** shows no inhibitory effect on the growth of this mycobacterial species.

**Table 1:** In vitro antimycobacterial activity and cytotoxicity of *R*-**2**, *S*-**2** and *rac*-**2**

	MIC_90_/µM *M. abscessus* ATCC 19977	MIC_90_/µM *M. smegmatis* mc2 155	EC_50_/µM^a^
*R*-**2**	6.25	0.78	> 30
*S*-**2**	> 100	> 100	> 30
*rac*-**2**	25	1.56	> 30

^a^RSB assay against the cell lines A375, HT29, MCF-7, A2780 and NIH 3T3

The lower sensitivity of *M. abscessus* compared with *M. smegmatis* to antibiotic agents is described in the literature (Maurer et al. 2014). Various causes, such as lower cell wall permeability, enzymatic inactivation or structural differences in the target, can be responsible for this. An exact elucidation of the mechanism would have exceeded the scope of the investigation described here.

Cytotoxicity of *R*-**2**, *S*-**2** and *rac*-**2** against eukaryotic cells was investigated by means of a RSB assay, analyzing the effect of the compounds on a panel of mammalian cell lines. The cytotoxicity assay showed that neither of the enantiomers and the racemate of **2** had a cytotoxic effect up to a concentration of 30 µM against the cell lines A375 (melanoma), HT29 (colon cancer), MCF-7 (breast cancer) (Soule et al. 1973), A2780 (ovarian cancer) and NIH 3T3 (mouse fibroblast) used in the assay (Table 1).

## Conclusion

The title compound (**2**) is a promising lead candidate for a synthetic RNA polymerase inhibitor against various mycobacterial species. Racemization-free synthesis of both enantiomers from Boc-protected d- or l-phenylalanine was achieved by exploiting the T3P^®^ and PyBOP^®^ reagents for amide coupling reactions, as confirmed by CD spectroscopy and chiral HPLC. This synthetic route avoids the need for subsequent separation of the enantiomers. X-ray crystallography of *rac*-**2** revealed a crystal and molecular structure of a compound of this class for the first time, as far as we are able to ascertain. Structural knowledge should be useful for further exploration of **2** and derivatives in medicinal chemistry. We observed different activities for both enantiomers of **2** against *M. smegmatis* and against the pathogenic *M. abscessus*, underlining the importance of absolute configuration for activity. Only the *R* enantiomer (*R*-**2**), derived from the non-proteinogenic amino acid d-phenylalanine shows antimycobacterial activity. No cytotoxicity against five mammalian cell lines was observed. The results of the present work encourage optimization of antimycobacterial activities (*i. e.* MIC < 1 µM) and pharmacokinetic properties of *N*α-aroyl-*N*-aryl-phenylalanine amides.

### Supplementary material

NMR and UV spectra for **2**, DSC analysis of *R*-**2** and in vitro growth inhibition activity of *R*-**2**, *S*-**2** and *rac*-**2** against *M. abscessus* ATCC 19977 and *M. smegmatis* mc2 155. CCDC 2049487 contains the supplementary crystallographic data for this paper. These data can be obtained free of charge from the Cambridge Crystallographic Data Centre via http://www.ccdc.cam.ac.uk/structures.

## Supplementary Information

Below is the link to the electronic supplementary material.

Supplementary file1 (PDF 115 KB)

Supplementary file2 (DOCX 3303 KB)

## Data Availability

Supplementary crystallographic data including reflection files have been deposited with the Cambridge Crystallographic Data Centre.
