# Supplementary material for: Racemization-free synthesis of Nα-2-thiophenoyl-phenylalanine-2-morpholinoanilide enantiomers and their antimycobacterial activity
Source: Amino Acids. 2021 Jul 14;53(8):1187–96. doi: 10.1007/s00726-021-03044-1 (PMC8325651; doi:10.1007/s00726-021-03044-1)
Supplement: Supplementary file 2 — Supplementary file2 (DOCX 3303 KB) [file 726_2021_3044_MOESM2_ESM.docx]

Supplementary material for

**Racemization-free synthesis of *N*α-2-thiophenoyl-phenylalanine-2-morpholinoanilide enantiomers and their antimycobacterial activity**

Lea Mann, Markus Lang, Philipp Schulze, Jan Henrik Halz, René Csuk, Sophie Hoenke, Rüdiger W. Seidel, Adrian Richter

**Figure S1** ^1^H NMR (400 MHz) of *rac*-**2** in CDCl_3_.

**Figure S2** ^13^C NMR (101 MHz) of *rac*-**2** in CDCl_3_.

**Figure S3** ^1^H NMR (400 MHz) of *S*-**2** in CDCl_3_.

**Figure S4** APT ^13^C NMR (101 MHz) of *S*-**2** in CDCl_3_.

**Figure S5** ^1^H NMR (400 MHz) of *R*-**2** in CDCl_3_.

**Figure S6** APT ^13^C NMR (101 MHz) of *R*-**2** in CDCl_3_.


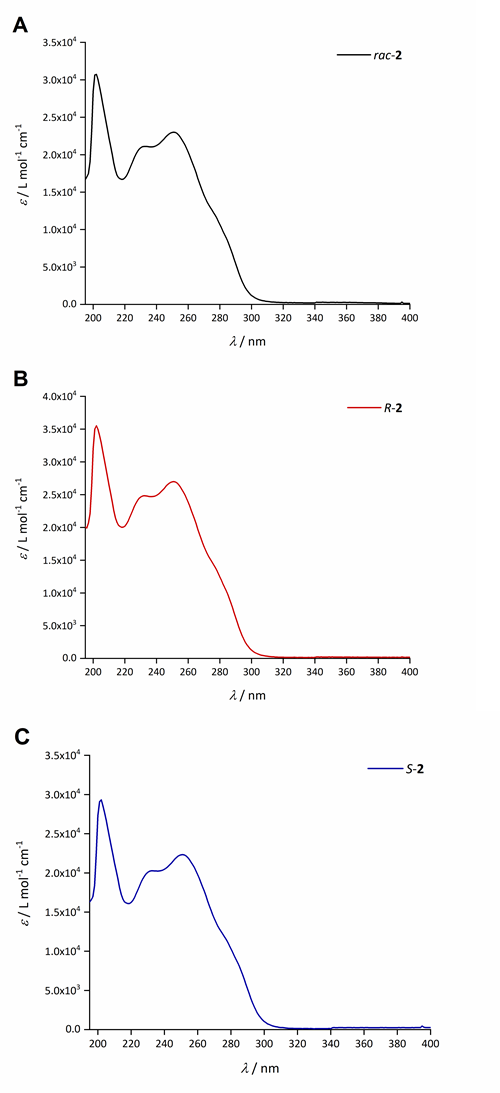


**Figure S7** UV-Vis spectra of (**A**) *rac*-**2** (*c* = 1.102 × 10^−5^ mol L^−1^)*,* (**B**) *R*-**2** (*c* = 9.18 × 10^−6^ mol L^−1^) and (**C**) *S-***2** (*c* = 9.18 × 10^−6^ mol L^−1^) in methanol.


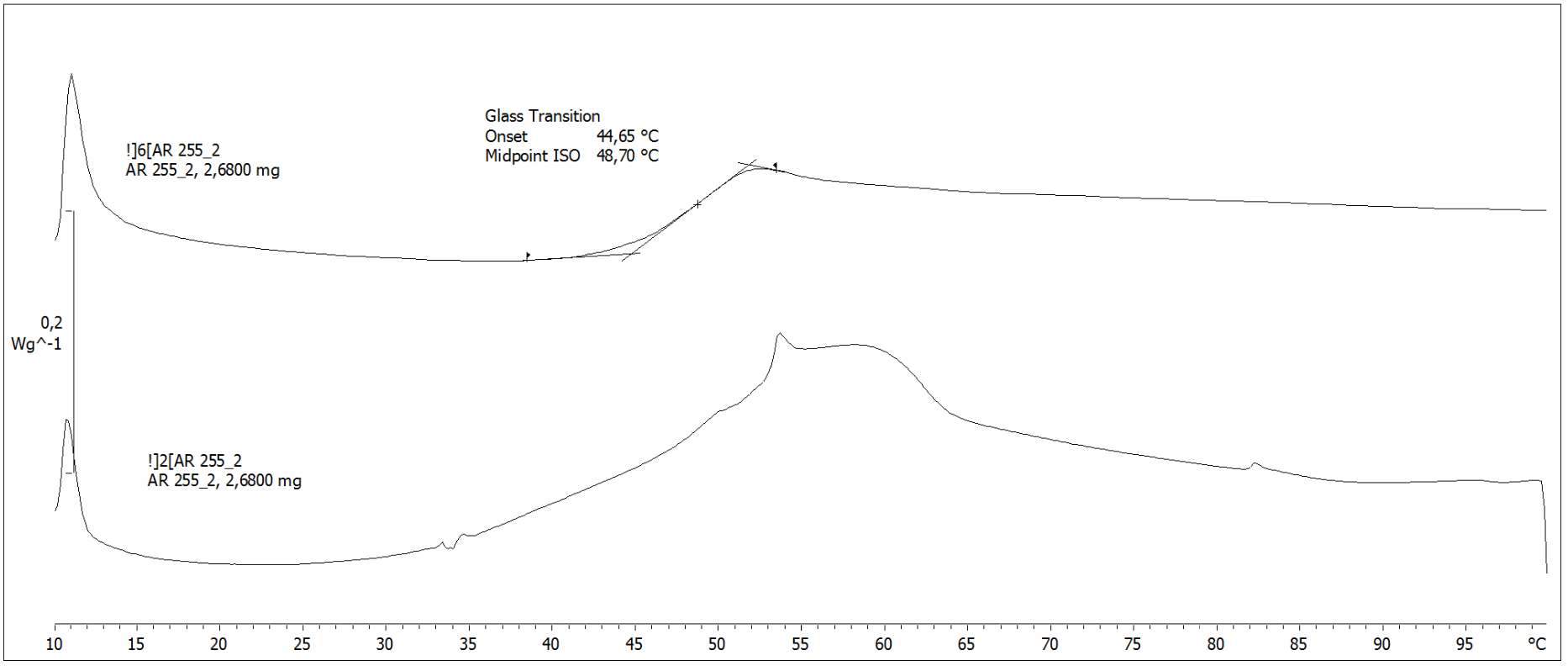


**Figure S8** DSC scans (heating rate 10 K min^−1^) of *R*-**2** (isolated from chloroform); the first scan (bottom line) shows evaporation of some residual solvent.

|  |  |  |
| --- | --- | --- |
|  |  |  |

**Figure S9** *In vitro* growth inhibition activity of *R*-**2**, *S*-**2** and *rac*-**2** against *M. abscessus* ATCC 19977 (**A**-**C**) and *M. smegmatis* mc2 155 (**D**-**F**) (AMK = amikacin)

**Figure S10 A** Chiral HPLC chromatogram of **1a (***t*_R_ =6.0 min), **B** HPLC separation of **1a** and its enantiomer (Chiralpak IC-3, acetonitrile/water + 0.1% TFA 30:70)
